# History of cannabis use and cognitive function in older adults: findings from the UK biobank

**DOI:** 10.1093/ageing/afaf319

**Published:** 2025-11-04

**Authors:** Sharon R Sznitman, Shiraz Vered, Galit Weinstein

**Affiliations:** School of Public Health, University of Haifa, Haifa, Israel; Institute of Primary Health Care (BIHAM), University of Bern, Switzerland; School of Public Health, University of Haifa, Haifa, Israel; School of Public Health, University of Haifa, Haifa, Israel

**Keywords:** aging, cannabis, cognitive decline, UK biobank, longitudinal effects, older people

## Abstract

**Background:**

Cannabis is a commonly used psychoactive drug, but its cognitive effects remain unclear, particularly in older adults. This study examined associations between past and present cannabis use and cognitive function among dementia-free older adults.

**Methods:**

Cross-sectional and longitudinal data were drawn from the UK Biobank, including adults aged ≥60 years. Cannabis use patterns were self-reported, and cognitive function was assessed via computerized tests of attention, executive function, processing speed, visual memory and working memory. Multivariable linear regression models adjusted for demographic, health and lifestyle-related covariates.

**Results:**

Cross-sectional analyses included 67 713 participants; longitudinal analyses included 52 002 participants with two cognitive assessments (mean age 67.2 ± 4.4 years; 46.1% male). Lifetime cannabis users (17%) performed better across all cognitive domains: attention (B = 0.071), executive function (B = 0.047), processing speed (B = 0.363), visual (B = 0.062) and working memory (B = 0.181). Current use was associated with better working memory (B = 0.169). Mixed and contradictory results were found for early onset, duration and frequency of use with cognitive outcomes. Longitudinally, past use was associated with less decline in executive function, while longer duration of use predicted steeper decline in processing speed.

**Conclusions:**

Cannabis use is not uniformly harmful to cognition in older adults. Past use was linked to better performance and slower decline in some cognitive domains. However, specific usage patterns, such as longer duration, were associated with poorer outcomes in other domains. These findings highlight the need for further research to clarify underlying mechanisms and guide evidence-based recommendations regarding cannabis use in aging populations.

## Key Points

- Cannabis use in older adults is not uniformly associated with cognitive decline; former users showed better cognitive perform.
- Longitudinal data suggest that past cannabis use is linked to slower decline in executive function over time.
- Longer duration of cannabis use was associated with faster decline in processing speed in older age.
- Early-onset and regular use were linked to mixed cognitive outcomes, suggesting domain-specific effects.
- These findings highlight the complex relationship between cannabis use and cognition in aging populations, warranting further.

## Introduction

The relationship between cannabis use and cognitive performance, particularly in aging populations, requires thorough examination due to increasing cannabis use among older adults [1–3], and its potential effects on age-related cognitive changes [4]. Studies on cannabis’s effects on long-term cognitive performance have focused on younger populations. Research shows frequent cannabis users perform worse on cognitive tests than infrequent or non-users, especially in verbal learning and memory [5–10]. However, a meta-analysis found associations between cannabis use and cognitive performance inconclusive [9], with some noting the relationship may not be clinically significant [6, 7]. Moreover, cognitive impairments from cannabis use typically diminish after abstinence [6, 8].

Studies on middle-aged individuals (average age ~40 years) have confirmed findings from younger samples regarding cognitive impairment from cannabis use [11]. While memory impairment findings are consistent, those for other cognitive domains in this age group remain inconsistent [11]. Notably, a study showed a positive correlation between self-reported cannabis use at age 42 years and better performance on cognitive assessments, including global cognitive function, memory and executive function, at age 50 years [12]. However, effect sizes were modest. No association with cognitive function was found between individuals with no use history and current cannabis users.

Beyond middle age, findings on cannabis consumption and cognitive function remain limited and inconsistent [4]. A study of 26,399 U.S. adults aged 50+ years showed past-year cannabis use was associated with higher rates of subjective memory complaints versus non-use, but no difference for past month use [13]. Three small cross-sectional studies of older adults (mean ages ~65–70 years) yielded contrasting results [14–16]. One found no significant differences in cognitive performance between current users and abstainers [14]. However, when categorized by duration, long-term use was associated with poorer processing speed and executive function compared to no use, while short-term use was not [15]. Another study [16] found no significant difference between older adults (mean age 66.6 years) with heavy cannabis use history but abstinent for an average of 28.7 years and controls without use, on neuropsychological domains of encoding and delayed memory, processing speed and executive function. Another study [17] found no significant differences in cognitive performance between older (mean age 61.4 years) medical cannabis licensed and non-licensed patients with chronic pain across cognitive domains.

Additionally, neuroimaging findings suggest frequent/prolonged cannabis use may be associated with smaller brain volumes in older adults [18], and preclinical studies indicate that age may influence how cannabis affects the brain, as low THC dose impaired function in young animals but improved cognition and brain structure in older mice [4, 19, 20]. Combined, this demonstrates the need to further clarify cannabis’s impact on cognition in aging populations. Current evidence comes from small, cross-sectional studies [14–17], necessitating larger longitudinal research to establish long-term effects in older populations. This research is crucial given increased cannabis use in this age group [1] and cognitive decline associated with normal aging [21]. While age, genetic predisposition and family background are primary predictors of cognitive deterioration and dementia, various modifiable lifestyle factors affect cognitive well-being in older individuals [22]. Cannabis use may be one such factor, but remains inadequately explored.

To advance this research field, we utilized data from the UK Biobank (UKBB) to investigate whether cannabis use (past and current) and patterns of use (age of onset, frequency and duration of use) are associated with cognitive performance cross-sectionally and longitudinally in a large sample of participants aged 60 years and older.

## Methods

### Study sample

The UKBB contains data on 500 000 healthy community members aged over 40, enlisted across Great Britain between 2006–2010 [23]. Details regarding protocols are accessible online [24]. Cognitive performance data were collected between 2014–2019, with assessments administered online or on-site. Cannabis use information was gathered through self-reported questionnaires between 2016–2017 and 2022–2023.

**Figure 1:**
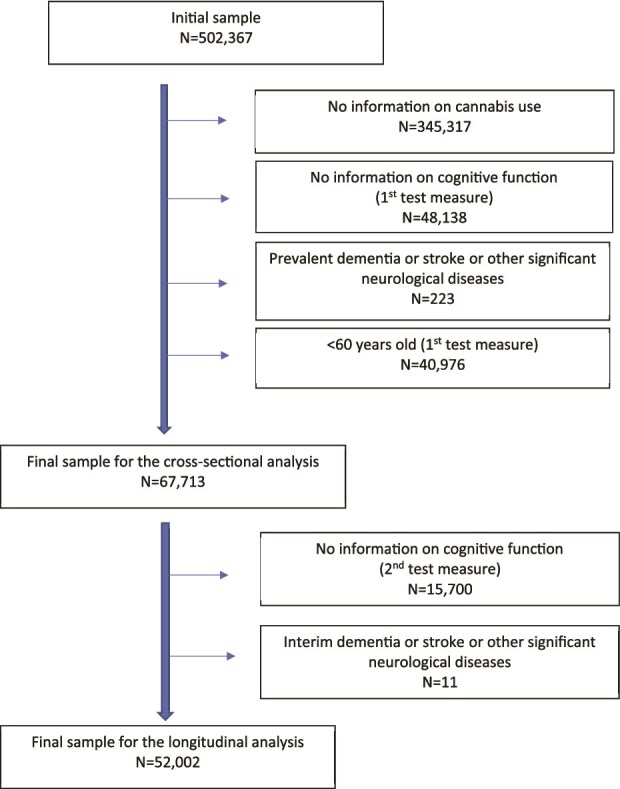
Flow chart of the study sample.

Figure 1 shows the initial cohort of 502 367 participants. After excluding those lacking data on cannabis use (N = 345,317) and cognitive performance (N = 48,138), and subjects with pre-existing dementia, stroke or neurological conditions and under age 60 at first cognitive assessment (N = 40,976), the final sample was 67,713.

For longitudinal analyses, we excluded 15 700 participants without second cognitive assessment data and 11 who developed interim dementia, stroke, or neurological diseases. This left 52 002 individuals. The UKBB received ethical approval from Northwest Multi-Centre Research Ethics Committee (11/NW/0382). All participants provided informed consent. The present study was conducted under UKBB project ID number 65753 [24].

### Cannabis use assessment

Participants were classified as no lifetime use or lifetime use. Lifetime users were categorized as current (use within the past year) or former (last use over a year prior). Frequency during the period of most regular use was coded: 1 = less than once a month, 2 = monthly but not weekly, 3 = weekly but not daily, 4 = daily. Regular use was defined as > once monthly (categories 2–4), with category 1 as the reference. Early onset (≤17 years at first use) and age at first use were recorded. Duration was calculated in years and as short-term (<5 years) or long-term (≥5 years). For former users we also included a measure of number of years abstinent.

### Cognitive assessment

Brief cognitive tests were administered via automated touchscreen without supervision were developed for UKBB or adapted for automated assessments [27] and included: (i) attention and executive function (Trail-making Tests A and B, completion time in seconds, log-transformed to normalize distributions), (ii) processing speed (Symbol Digit Test, correct matches), (iii) visual memory (pair matching test, incorrect matches) and (iv) working memory (numeric memory test, maximum digits remembered). Higher scores indicated better performance. Visual and working memory tests were unavailable at follow-up and excluded from longitudinal analyses.

### Covariates

Covariates, assessed before initial cognitive testing, included education (college/some college or lower), socioeconomic status (Townsend index), tobacco and alcohol use (never, former, current), history of alcohol or other substance addiction, moderate physical activity (≥10 minutes, days/week), obesity (BMI ≥30) and comorbidities (ischemic heart disease, hypertension, diabetes, mood disorders; ICD-10).

### Statistical analysis

For continuous variables, results are presented as mean and standard deviation or median (q1, q3) for skewed distributions. Groups were compared using T-test or Wilcoxon test for non-parametric data. Categorical variables were expressed as frequencies and percentages, with comparisons using chi-square test.

Cross-sectional and longitudinal associations between cannabis use patterns and cognitive performance were examined using multiple linear regression models. Three models were used: first comparing no use versus lifetime use; second with former and current use as predictors (no lifetime use as reference); third excluding abstainers with former use as reference and current use as predictor. To further examine cannabis use-cognitive performance relationships, various models were conducted for former and current use subgroups, each using one of the following predictors: frequency of use, early onset, age at onset, duration of use (in years and long vs. short). Models accounted for all covariates except mood disorders due to low prevalence.

For longitudinal analysis of cannabis use and cognitive performance, models used difference scores between cognitive measures as dependent variables, with first measures and inter-assessment duration as covariates. The same predictors and covariates were used as in cross-sectional analysis. Results were significant at *P* < .05, using SAS software version 9.4.

## Results

### Sample descriptives

Table 1 displays the demographic characteristics of the entire sample and subgroups based on cannabis use patterns. The average age was 67.2 ± 4.4 years, with 31 204 (46.1%) being men. Of the entire cohort (N = 67,713), 11 831 individuals (17%) reported having used cannabis at least once in their lives. Among these, 502 (4%) had used cannabis in the past year (current use). In comparison to those who never used cannabis, individuals who reported lifetime cannabis use were more likely to be men, younger, of higher socioeconomic status, more likely to report current or former tobacco and alcohol use, lifetime substance use addition and less prone to obesity (all *P* < .001).

**Table 1:** Sample characteristics—overall, by cannabis use and by pattern of use.

		Total N = 67,713 (100%)	Non-use vs. use			Former vs. current use		
Characteristics			No-use N = 55,882 (83%)	Use N = 11,831 (17%)	p-value	Former use N = 11,329 (96%)	Current use N = 502 (4%)	p-value
Age, y		67.2 ± 4.4	67.5 ± 4.4	65.5 ± 4.0	<0.001	65.5 ± 4.0	64.7 ± 3.4	<0.001
Men		31,204 (46.1)	24,863 (44.5)	6341 (53.6)	<0.001	6025 (53.2)	316 (63.0)	<0.001
Education (college/some college)		61,787 (91.6)	50,379 (90.5)	11,408 (96.7)	<0.001	10,938 (96.8)	470 (94.6)	0.007
Tobacco smoking status	Never	37,681 (55.7)	34,210 (61.3)	3471 (29.4)	<0.001	3401 (30.1)	70 (14.1)	<0.001
	Past	26,675 (39.5)	19,545 (35.0)	7130 (60.4)		6889 (60.9)	241 (48.4)	
	Current	3251 (4.8)	2066 (3.7)	1204 (10.2)		1017 (9.0)	187 (37.6)	
Alcohol use	Never	2116 (3.1)	2066 (3.7)	50 (0.4)	<0.001	48 (0.4)	2 (0.4)	0.800
	Past	2052 (3.0)	1659 (3.0)	393 (3.3)		379 (3.4)	14 (2.8)	
	Current	63,500 (93.8)	52,126 (93.3)	11,374 (96.3)		10,891 (96.2)	483 (96.8)	
Socioeconomic status		−2.0 ± 2.7	−2.2 ± 2.6	−1.0 ± 3.0	<0.001	−1.1 ± 3.0	0.2 ± 3.3	<0.001
Physical activity		4.0 [2.0–6.0]	4.0 [2.0–6.0]	4.0 [2.0–6.0]	0.897	4.0 [2.0–6.0]	4.0 [2.0–5.0]	0.435
Obesity		12,939 (19.1)	10,841 (19.4)	2098 (17.8)	<0.001	2010 (17.8)	88 (17.5)	0.892
Ischemic Heart Disease		387 (0.6)	334 (0.6)	54 (0.5)	0.050	50 (0.4)	3 (0.6)	0.608
Hypertension		2061 (3.0)	1716 (3.1)	345 (2.9)	0.374	337 (3.0)	8 (1.6)	0.072
Diabetes		1235 (1.8)	1015 (1.8)	220 (1.9)	0.750	209 (1.8)	11 (2.2)	0.574
Mood disorders		644 (1.0)	522 (0.9)	122 (1.0)	0.323	122 (1.1)	0 (0.0)	0.019
Lifetime substance use addiction		3368 (5.0)	2134 (3.8)	1234(10.6)	<0.001	1141 (10.3)	93 (19.3)	<0.001
Frequently of cannabis use								
Less than once a month		-	-	7698 (67.4)	-	7542 (69.0)	156 (31.5)	<0.001
Once a month or more, but not every week		-	-	1350 (11.8)		1284 (11.8)	66 (13.3)	
Once a week or more, but not every day		-	-	1816 (15.9)		1679 (15.4)	137 (27.6)	
Every day		-	-	557 (4.9)		420 (3.8)	147 (27.6)	
Regular use (more than once a month)		-	-	3723 (32.6)	-	3383 (31.0)	340 (68.6)	<0.001
Age at first cannabis use		-	-	20.0 [18.0–24.0]	-	20.0 [18.0–24.0]	20.0 [18.0–25.0]	0.025
Early onset (first cannabis use prior to 17 years of age)		-	-	1051 (13.6)	-	975 (13.2)	76 (22.1)	<0.001
Duration of use, y		-	-	5.0 [1.0–15.0]	-	5.0 [1.0–13.0]	45.0 [41.0–47.0]	<0.001
Long duration of use (duration of use was >5 years)		-	-	3873 (52.8)	-	3546 (50.7)	327 (96.8)	<0.001
Duration of cannabis use abstinence		-	-	-	-	38.0 [25.0–43.0]	-	-

Continuous values are reported as Mean ± SD or Median [q1-q3] and dichotomous values are reported as N (%).

Obesity was defined as body mass index ≥30; Physical activity was defined as number of days per week of moderate physical activity >10 min; Current use defined if they had used cannabis within the year before the initial cognitive assessment; Regular use defined as use of cannabis more than once a month; Early onset was defined as reported initial cannabis use prior to 17 years of age, late onset was defined as reported initial cannabis use at age 17 or later; Long duration of use was defined when duration of use was >5 years.

Compared to participants who reported former cannabis use, those who reported current use were more likely to report more frequent/regular use (68.6% vs. 31%), early onset of use (22.1% vs. 13.2%) and extended duration of use (duration >5 years, 96.8% vs. 50.7%; mean years used: 45 vs. 5).

### Cannabis use and cognitive performance

Multivariable analyses comparing participants with and without lifetime cannabis use (Table 2, panel A) showed that after controlling for covariates, lifetime cannabis use was associated with better performance on all cognitive tests (*P* < .001) (attention: B = 0.071 ± 0.005; executive function: B = 0.047 ± 0.004; processing speed: B = 0.363 ± 0.052; visual memory: B = 0.062 ± 0.014; working memory: B = 0.181 ± 0.016). A more nuanced examination contrasting lifetime abstinence with former and current use (Table 2, Panel B) revealed the same overall results for participants with former use, whereas current use was only associated with better working memory than never use (working memory: B = 0.169 ± 0.070; *P* = .016). When comparing current use to former use (Table 2, panel C), the results showed that current use was associated with poorer performance in attention (B = −0.099 ± 0.020; *P* < .001), executive function (B = −0.056 ± 0.017; *P* = .001) and processing speed (B = −0.496 ± 0.223; *P* = .026).

**Table 2:** Results from multivariable regression analyses showing the associations of cannabis use with cognitive function

Cannabis use	Attention B (SE) N = 57,998	Executive function B (SE) N = 57,499	Processing speed B (SE) N = 64,205	Visual memory B (SE) N = 66,400	Working memory B (SE) N = 61,059
Panel A – comparing ever use to no use					
No use	0 (ref.)	0 (ref.)	0 (ref.)	0 (ref.)	0 (ref.)
Ever use	**0.071 (0.005)** ***P* < .001**	**0.047 (0.004)** ***P* < .001**	**0.363 (0.052)** ***P* < .001**	**0.062 (0.014)** ***P* < .001**	**0.181 (0.016)** ***P* < .001**
Panel B – comparing former use and current use to never use					
No use	0 (ref.)	0 (ref.)	0 (ref.)	0 (ref.)	0 (ref.)
Former use	**0.074 (0.005)** ***P* < .001**	**0.049 (0.004)** ***P* < .001**	**0.381 (0.052)** ***P* < .001**	**0.064 (0.015)** ***P* < .001**	**0.181 (0.016)** ***P* < .001**
Current use	−0.032 (0.020) *P* = .118	−0.009 (0.017) *P* = .576	−0.125 (0.222) *P* = .574	−0.005 (0.062) *P* = .927	**0.169 (0.070)** ***P* = .016**
Panel C – comparing current use to former use					
Former use	0 (ref.)	0 (ref.)	0 (ref.)	0 (ref.)	0 (ref.)
Current use	**−0.099 (0.020)** ***P* < .001**	**−0.056 (0.017)** ***P* = .001**	**−0.496 (0.223)** ***P* = .026**	−0.064 (0.062) *P* = .300	−0.005 (0.069) *P* = .943

SE, Standard error; Attention, Trail Making A; Executive function, Trail Making B; Processing speed, Symbol digit; Visual memory, pairs matching; Working memory, Numeric memory; Current use defined if they had used cannabis within the year before the initial cognitive assessment.

Models adjusted for age, sex, education, socioeconomic status, tobacco smoking, alcohol use, lifetime substance use addiction, obesity, physical activity and history of ischemic heart disease, hypertension and diabetes.

Bold values indicate statistical significance (*P* < .05).

In terms of cannabis use patterns, among participants with former use, having used cannabis more than once per month was associated with better attention (once a month, but not weekly: B = 0.028 ± 0.012; *P* = .028; more than once a month vs. less frequently: B = 0.024 ± 0.009; *P* = .007). Older age at initiation was associated with worse attention (B = −0.004 ± 0.0006; *P* < .001) and worse executive function (B = −0.002 ± 0.0001; *P* < .001) and compared to early onset of first use, late onset was associated with worse attention (B = −0.094 ± 0.014; *P* < .001) and visual memory (B = −0.116 ± 0.046; *P* = .011), but better working memory (B = 0.140 ± 0.049; *P* = .005). Furthermore, longer duration of use was associated with slower processing speed (measured as categorical >5 years: B = −0.281 ± 0.110, *P* = .011; measured as continuous (years): B = −0.011 ± 0.005; *P* = .027) but better attention ((years): B = 0.001 ± 0.0001; *P* = .018).

Among current users, regular use (monthly use vs. less) was associated with better attention (B = 0.084 ± 0.039; *P* = .033) whereas late onset (vs. early onset) was associated with better visual memory (B = 0.351 ± 0.171; *P* = .041) (Table 3).

**Table 3:** Results from multivariable regression analyses showing the associations of pattern of cannabis use with cognitive function

Pattern of use		Attention B (SE)	Executive function B (SE)	Processing speed B (SE)	Visual memory B (SE)	Working memory B (SE)
Former use	N	9999	9943	10,837	11,137	10,478
	Less than once a month	0 (ref.)	0 (ref.)	0 (ref.)	0 (ref.)	0 (ref.)
	Once a month or more, but not every week	**0.028 (0.012)** ***P* = .028**	0.—9 (0.010) *P* = .382	0.148 (0.142) *P* = .297	0.020 (0.039) *P* = .613	0.021 (0.043) *P* = .627
	Once a week or more, but not every day	0.018 (0.011) *P* = .103	0.012 (0.009) *P* = .207	−0.049 (0.128) *P* = .701	0.033 (0.036) *P* = .355	0.033 (0.039) *P* = .392
	Every day	0.033 (0.021) *P* = .131	0.008 (0.017) *P* = .636	0.049 (0.241) *P* = .837	−0.012 (0.067) *P* = .849	0.044 (0.073) *P* = .544
	Not regular use	0 (ref.)	0 (ref.)	0 (ref.)	0 (ref.)	0 (ref.)
	Regular use	**0.024 (0.009)** ***P* = .007**	0.010 (0.007) *P* = .154	0.039 (0.099) *P* = .694	0.022 (0.028) *P* = .412	0.030 (0.030) *P* = .324
	Early onset	0 (ref.)	0 (ref.)	0 (ref.)	0 (ref.)	0 (ref.)
	Late onset	−0.094 (0.014) *P* < .001	−0.022 (0.012) *P* = .062	0.139 (0.161) *P* = .388	**−0.116 (0.046)** ***P* = .011**	**0.140 (0.049)** ***P* = .005**
	Age at cannabis use initiation	**−0.004 (0.0006)** ***P* < .001**	**−0.002 (0.0001)** ***P* < .001**	−0.007 (0.007) *P* = .353	−0.003 (0.002) *P* = .162	−0.003 (0.002) *P* = .148
	Short duration	0 (ref.)	0 (ref.)	0 (ref.)	0 (ref.)	0 (ref.)
	Long duration	0.002 (0.010) *P* = .806	−0.012 (0.008) *P* = .143	**−0.281 (0.110) *P* = .011**	−0.004 (0.031) *P* = .991	0.018 (0.034) *P* = .590
	Duration of use	**0.001 (0.0001)** ***P* = .018**	−0.0001 (0.0001) *P* = .185	**−0.011 (0.005) *P* = .027**	0.002 (0.001) *P* = .158	0.003 (0.002) *P* = .084

Current use	N	410	409	472	486	442
	Less than once a month	0 (ref.)	0 (ref.)	0 (ref.)	0 (ref.)	0 (ref.)
	Once a month or more, but not every week	0.099 (0.057) *P* = .087	0.029 (0.053) *P* = .587	−0.179 (0.732) *P* = .807	0.181 (0.190) *P* = .341	−0.015 (0.232) *P* = .950
	Once a week or more, but not every day	0.078 (0.047) *P* = .097	0.029 (0.043) *P* = .493	0.386 (0.606) *P* = .525	0.169 (0.157) *P* = .282	0.003 (0.191) *P* = .988
	Every day	0.081 (0.049) *P* = .103	0.036 (0.045) *P* = .430	−0.071 (0.641) *P* = .912	0.081 (0.167) *P* = .628	0.238 (0.201) *P* = .239
	Not regular use	0 (ref.)	0 (ref.)	0 (ref.)	0 (ref.)	0 (ref.)
	Regular use	**0.084 (0.039)** ***P* = .033**	0.032 (0.036) *P* = .380	0.098 (0.508) *P* = .850	0.142 (0.132) *P* = .283	0.080 (0.159) *P* = .613
	Early onset	0 (ref.)	0 (ref.)	0 (ref.)	0 (ref.)	0 (ref.)
	Late onset	−0.083 (0.056) *P* = .141	0.076 (0.051) *P* = .134	0.233 (0.746) *P* = .755	**0.351 (0.171)** ***P* = .041**	0.066 (0.228) *P* = .773
	Age at cannabis use initiation	−0.001 (0.002) *P* = .429	0.001 (0.002) *P* = .538	−0.021 (0.025) *P* = .413	0.007 (0.006) *P* = .266	0.002 (0.008) *P* = .856
	Duration of use	0.0001 (0.002) *P* = .694	−0.002 (0.002) *P* = .357	0.016 (0.025) *P* = .528	−0.007 (0.006) *P* = .224	−0.0001 (0.008) *P* = .881

SE, Standard error; Attention, Trail Making A; Executive function, Trail Making B; Processing speed, Symbol digit; Visual memory, pairs matching; Working memory, Numeric memory; Current use defined if they had used cannabis within the year before the initial cognitive assessment; Regular use defined as use of cannabis more than once a month; Early onset was defined as reported initial cannabis use prior to 17 years of age, late onset was defined as reported initial cannabis use at age 17 or later; Long duration of use was defined when duration of use was >5 years.

Models adjusted for age, sex, education, socioeconomic status, tobacco smoking, alcohol use, lifetime substance use addiction, obesity, physical activity and history of ischemic heart disease, hypertension and diabetes.

Bold values indicate statistical significance (*P* < .05).

### Cannabis use and cognitive decline

The longitudinal sample descriptions are available in Appendix 1, Supplementary Table S1. Of the participants who underwent two cognitive assessments (N = 52,002), 9468 (18%) reported lifetime cannabis use. Among these, 376 (4%) reported current use (Appendix 1, Supplementary Table S2). The mean duration between the first and second cognitive assessments was 5.7 ± 2.0 years.

**Figure 2:**
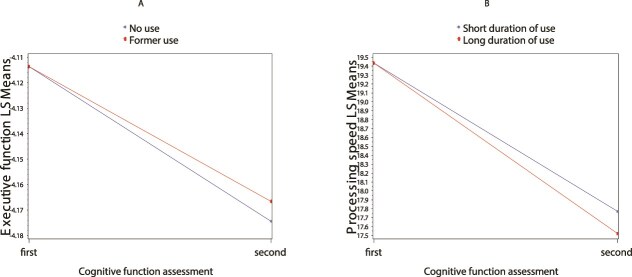
Significant associations of cannabis use with change in cognitive function over time. A: former use vs. no use with change in executive function; B: longer vs. short duration of use with change in processing speed, when former use.

Compared to participants without lifetime cannabis use, past cannabis use experience was associated with a decreased decline in executive function over time (ever use: B = 0.008 ± 0.004; *P* = .030), and this was true when comparing former use (B = 0.008 ± 0.004; *P* = .030), but not current use (B = 0.0004 ± 0.016; *P* = .774) with never use (Appendix 1, Supplement Table S3, fig. 2A). Among participants with past use, longer vs. short duration of cannabis use was associated with an increased decline in processing speed over time (B = −0.248 ± 0.118; *P* = .035; Appendix 1, Supplement Table S4, Figure 2B). Additionally, each year of use was associated with a statistically significant accelerated decline in processing speed (B = −0.019 ± 0.005; *P* < .001). Yet, among current users, pattern of cannabis use was not associated with any cognitive change (Appendix 1, Supplement Table S4).

## Discussion

Findings from our cross-sectional analyses show that participants with cannabis use experience performed better across all cognitive assessments compared to those without. These associations were primarily driven by participants with former use experience, while current use experience showed a more limited association, reaching significance only for working memory tasks. Similarly, our longitudinal analysis demonstrated a slower cognitive decline among former users, with no significant associations observed for current users. However, these null findings for current use should be interpreted with caution due to the small number of current users, which likely limited statistical power.

The observed positive relationship between lifetime cannabis use and cognitive performance across multiple cognitive domains may reflect the complex relationship between cannabis use during the life-span and cognition, due to the differential effects of the various cannabinoids and an effect-modification of individual-level characteristics including age and genetic predisposition [28]. Particularly, evidence points to distinct effects on the brain when cannabis is used in different ages. Indeed, some evidence from both preclinical and clinical studies suggests that cannabis may have beneficial effects on the brain when consumed in older ages [4, 29–31], and certain cannabinoids have shown neuroprotective effects [32, 33].

An alternative explanation is that our findings are a result of residual confounding. Notably, lifetime cannabis use was more prevalent among participants with a college education and higher socioeconomic status. These demographic characteristics typically correlate with employment in cognitively demanding occupations, potentially contributing to enhanced cognitive abilities in old age [34, 35]. In addition to residual confounding, other methodological limitations may contribute to the mixed findings observed in the literature and in our study. These include measurement error, such as recall bias or social desirability bias in self-reported cannabis use, as well as survival bias whereby individuals with the heaviest or most harmful patterns of use may have experienced adverse health outcomes or premature mortality, and thus are underrepresented in our sample.

It is also possible that the social and historical context of our study population provides additional insight into our findings. Our sample primarily comprised members of the baby boomer generation, born after 1945, a group that has historically demonstrated more liberal attitudes toward cannabis and higher rates of use than previous generations [1]. Among this generation, cannabis use may have become relatively normalized, reducing its association with social marginalization or risk behaviour. Consequently, individuals reporting lifetime cannabis use in this cohort may reflect a subgroup that is socially integrated and well-adjusted, rather than deviant. In this context, the observed positive association between cannabis use and cognitive function may reflect pre-existing differences in social-cognitive, executive and memory abilities, traits that support both greater social adjustment and the adoption of cannabis use behaviours that, within this generational milieu, are not stigmatized. A similar interpretation was made in a study among adolescents, where occasional cannabis use was associated with equivalent or slightly better cognitive ability compared to abstainers [36]. Further studies that include participants before and after cannabis initiation are needed to better understand these results.

It is also important to note that while comparing cannabis use to non-use provides insights, it may mask the complex relationship between cannabis and cognition. Examining specific patterns, including early onset, regular use and duration, offer a more granular understanding of the potential effects of cannabis on cognitive function. Our analysis of these patterns of use revealed counterintuitive relationships, challenging previous assumptions. First, in line with our main findings showing positive associations between cannabis use and cognition in general, we also found that among former users, regular use and earlier onset were associated with improved attention and visual memory. Additionally, past use was associated with a slower decline in executive function.

However, evidence for negative associations were also observed. Specifically, we found that former users who started using cannabis before the age of 17 had poorer working memory compared to those with later onset of use. Additionally, long duration of use was associated with slower processing speed and accelerated decline in processing speed over time. The possible detrimental effects of long periods of cannabis use are also implied by our observation that current vs. former cannabis use was associated with poorer cognitive outcomes in attention, executive function and processing speed, because participants who reported current use had significantly longer use durations compared to those with former use experience. These findings are consistent with existing literature suggesting that early onset, heavy and chronic use are linked to cognitive impairments in memory, attention and executive functions [37, 38]. Yet, while these results may highlight the detrimental effects of cannabis on the brain, they may also relate to a reciprocal relationship in which poor social adjustment relate to chronic and early onset cannabis use [39], which in turn, relate to further detrimental effects on the brain [40].

Our mixed results may also relate to cognitive domain-specific recovery after prolonged abstinence. The persistence or recovery of cognitive impairments across domains remains under-researched, with some studies indicating that deficits in verbal memory, attention and certain executive functions may persist [38]. Our findings suggest that early onset and prolonged use may be particularly detrimental for cognitive performance, harming working memory and processing speed in a way resistant to recovery. Since these findings are based on observational data, further experimental studies are needed to better understand the complex and potentially domain-specific long-term effects of cannabis use on cognition [41]. It is important to note that early onset, frequency and duration of use often overlap and may work synergistically to affect cognitive outcomes. Yet, due to high collinearity between these measures, we analysed their associations separately to avoid statistical confounding.

For current users, far fewer significant associations were found. Specifically, regular use in this group was associated with better attention whereas early onset was associated with poorer visual memory. While this may suggest that different use patterns are not as relevant to cognition in this sub-population compared to past users, it could also relate to lack of statistical power, as only 502 participants reported current use. Further research with larger samples of older adults with current use is necessary to draw more definitive conclusions about the relationship between cannabis use patterns and cognitive performance.

Our findings have implications for public health communication. While cognitive harms are often assumed among older cannabis users, our results suggest a more nuanced picture. Former use was linked to better performance in some domains and slower executive function decline, with no evidence of global harm, particularly among occasional or past users and not among current users. In contrast, long duration and early onset were associated with poorer outcomes in specific domains, such as working memory and processing speed. These findings may support balanced messaging that informs decisions, reduces age-related cannabis stigma and promotes clinician–patient dialogue on therapeutic cannabis. Clinician reluctance to prescribe medical cannabis to older adults is documented [42], as so is patient reluctance to discuss medical cannabis [43], despite evidence of benefits and limited harms in this group [44, 45]. Given the low NHS prescribing rates since legalization [46, 47], many UK clinicians may have limited experience with older therapeutic cannabis users.

### Strengths and limitations

Our research presents several innovative aspects. We employed data from a substantial sample, covering demographic, lifestyle and health factors, allowing us to eliminate many confounding variables. While the UKBB sample is not nationally representative, the data are not from convenience samples, enhancing generalizability. Repeated cognitive assessment enabled us to examine cannabis consumption’s relationship with cognitive changes over time.

However, certain limitations must be acknowledged. First, self-reported cannabis use may have introduced recall bias. Given participants were free from dementia, this bias is likely non-differential, underestimating true associations. Second, we could not distinguish recreational from medical cannabis use, though it is likely most reported use was non-medical. Medical cannabis was legalized in the UK in 2018, but NHS uptake has been minimal, only 3–5 prescriptions in the first five years, rising to ~5000 by 2023 [46, 47]. While private prescriptions are more common, older adults are underrepresented [44, 48]. Given the timing of data collection and limited access to prescribed cannabis, medical use is unlikely to have substantially confounded our results.

A third limitation is the limited granularity of our cannabis use measures. While our approach follows conventions in large-scale epidemiological studies [25, 26], the lack of detail on dose, cannabinoid composition and mode of administration limits our ability to assess dose–response associations. As cognitive effects may vary with these factors [37, 49–51], future research should include detailed cannabis regimen data to clarify conditions under which cannabis is linked to harm or resilience in later-life cognition.

Fourth, a range of concurrent validity coefficients has been observed for the UKBB cognitive test battery, while moderate to good test–retest reliability has been demonstrated [27]. Consequently, cognitive assessments may not be fully valid, potentially introducing non-differential information bias that could underestimate true associations. Fifth, attrition from the second cognitive assessment may relate to both cannabis use and cognition, with retained participants differing demographically from those completing one assessment. Additional research is needed to investigate this issue. Lastly, despite adjustment for covariates, residual confounding may persist.

## Conclusion

The findings of this study provide novel evidence on the complex relationship between cannabis use and cognitive function in older adults. As cannabis use increases among older populations, understanding its cognitive effects becomes crucial for public health. These results offer preliminary evidence that cannabis use may not be uniformly detrimental to cognitive health in aging. However, more research is needed to fully elucidate both the risks and potential benefits of cannabis use in older adults.

## Supplementary Material

aa-25-1440-File004_afaf319
