# Supplementary material for: History of cannabis use and cognitive function in older adults: findings from the UK biobank
Source: Age Ageing. 2025 Nov 4;54(11):afaf319. doi: 10.1093/ageing/afaf319 (PMC12586318; doi:10.1093/ageing/afaf319)
Supplement: aa-25-1440-File004_afaf319 [file aa-25-1440-file004_afaf319.docx]

**History of cannabis use and cognitive function in older adults: findings from the UK Biobank**

**Appendix 1:**

**Content list:**

1. **Supplement Table 1.** Sample characteristics of participants who completed only the first cognitive function assessment compared to participants who completed two cognitive function assessments
2. **Supplement Table 2.** Sample characteristics for participants who underwent two cognitive function measures – overall, by cannabis use and by pattern of use
3. **Supplement Table 3.** Results from multivariable regression analyses showing the associations of pattern of cannabis use with change in cognitive function over time
4. **Supplement Table 4.** Results from multivariable regression analyses showing the associations of pattern of cannabis use with change in cognitive function over time

**Supplement Table 1.** Sample characteristics of participants who completed only the first cognitive function assessment compared to participants who completed two cognitive function assessments

| **Variables** | | **Participants who completed only the first cognitive function assessment N=15,711 (23%)** | **Participants who completed two cognitive function assessments N=52,002 (77%)** | **p-value** |
| --- | --- | --- | --- | --- |
| Age, y | | 67.8 ± 4.4 | 67.0 ± 4.4 | <0.001 |
| Men | | 7630 (48.6) | 23574 (45.3) | <0.001 |
| Education (college/some college) | | 13844 (88.6) | 47943 (92.5) | <0.001 |
| Tobacco smoking status | Never | 8369 (53.4) | 29312 (56.5) | <0.001 |
|  | Past | 6387 (40.7) | 20288 (39.1) |  |
|  | Current | 928 (5.9) | 2323 (4.5) |  |
| Alcohol use status | Never | 542 (3.5) | 1574 (3.0) | <0.001 |
|  | Past | 554 (3.5) | 1498 (2.9) |  |
|  | Current | 14601 (93.0) | 48899 (94.1) |  |
| Socioeconomic status | | -1.9 ± 2.7 | -2.0 ± 2.7 | 0.079 |
| Physical activity | | 4.0 [2.0-6.0] | 4.0 [2.0-6.0] | 0.109 |
| Obesity | | 3435 (21.9) | 9504 (18.3) | <0.001 |
| Ischemic Heart Disease | | 85 (0.5) | 302 (0.6) | 0.562 |
| Hypertension | | 444 (2.8) | 1617 (3.1) | 0.070 |
| Diabetes | | 336 (2.1) | 899 (1.7) | <0.001 |
| Mood disorders | | 142 (0.9) | 502 (1.0) | 0.486 |

Continuous values are reported as Mean ±SD or Median [q1-q3] and dichotomous values are reported as N (%).

Obesity was defined as body mass index ≥30; Physical activity was defined as number of days per week of moderate physical activity >10 min

**Supplement Table 2.** Sample characteristics for participants who underwent two cognitive function measures – overall, by cannabis use and by pattern of use

|  | | **Total N=52,002 (100%)** | **Non-use vs. use** | | | **Former vs. current use** | | |
| --- | --- | --- | --- | --- | --- | --- | --- | --- |
| **Characteristics** | |  | **No-use N=42,534 (82%)** | **Use**  **N=9,468 (18%)** | **p-value** | **Former use N=9,092  (96%)** | **Current use**  **N= 376  (4%)** | **p-value** |
| Age, y | | 67.0 ± 4.4 | 67.3 ± 4.4 | 65.4 ± 4.0 | <0.001 | 65.4 ± 4.0 | 64.3 ± 3.3 | <0.001 |
| Men | | 23574 (45.3) | 18572 (43.7) | 5002 (52.8) | <0.001 | 4773 (52.5) | 229 (60.9) | 0.001 |
| Education (college/some college) | | 47943 (92.5) | 38791 (91.5) | 9152 (96.9) | <0.001 | 8800 (97.0) | 352 (94.9) | 0.022 |
| Tobacco smoking status | Never | 29312 (56.5) | 26498 (62.4) | 2814 (29.8) | <0.001 | 2761 (30.4) | 53 (14.2) | <0.001 |
|  | Past | 20288 (39.1) | 14576 (34.3) | 5712 (60.5) |  | 5526 (60.9) | 186 (49.7) |  |
|  | Current | 2323 (4.5) | 1401 (3.3) | 922 (9.8) |  | 787 (8.7) | 135 (36.1) |  |
| Alcohol use | Never | 1574 (3.0) | 1534 (3.6) | 40 (0.4) | <0.001 | 38 (0.4) | 2 (0.5) | 0.683 |
|  | Past | 1498 (2.9) | 1203 (2.8) | 295 (3.1) |  | 286 (3.2) | 9 (2.4) |  |
|  | Current | 48899 (94.1) | 39776 (93.6) | 9123 (96.5) |  | 8760 (96.4) | 363 (97.1) |  |
| Socioeconomic status | | -2.0 ± 2.7 | -2.2 ± 2.6 | -1.0 ± 3.0 | <0.001 | -1.1 ± 3.0 | 0.1 ± 3.2 | <0.001 |
| Physical activity | | 4.0 [2.0-6.0] | 4.0 [2.0-6.0] | 4.0 [2.0-6.0] | 0.264 | 4.0 [2.0-6.0] | 4.0 [2.0-6.0] | 0.646 |
| Obesity | | 9504 (18.3) | 7908 (18.6) | 1596 (16.9) | <0.001 | 1530 (16.9) | 66 (17.6) | 0.722 |
| Ischemic Heart Disease | | 302 (0.6) | 260 (0.6) | 42 (0.4) | 0.052 | 40 (0.4) | 2 (0.5) | 0.793 |
| Hypertension | | 1617 (3.1) | 1345 (3.2) | 272 (2.9) | 0.142 | 267 (2.9) | 5 (1.3) | 0.068 |
| Diabetes | | 899 (1.7) | 733 (1.7) | 166 (1.8) | 0.840 | 159 (1.8) | 7 (1.9) | 0.870 |
| Mood disorders | | 502 (1.0) | 403 (1.0) | 99 (1.1) | 0.377 | 99 (1.1) | 0 (0.0) | 0.042 |
| Lifetime substance use addiction | | 2525 (4.9) | 1562 (3.7) | 963(10.3) | <0.001 | 892 (10.0) | 71 (19.6) | <0.001 |
| Frequently of cannabis use | |  |  |  |  |  |  |  |
| Less than once a month | | - | - | 6166 (67.3) | - | 6053 (68.9) | 113 (30.5) | <0.001 |
| Once a month or more, but not every week | | - | - | 1075 (11.7) | - | 1023 (11.6) | 52 (14.0) |  |
| Once a week or more, but not every day | | - | - | 1469 (16.0) | - | 1366 (15.5) | 103 (27.8) |  |
| Every day | | - | - | 453 (4.9) | - | 350 (4.0) | 103 (27.8) |  |
| Regular use (more than once a month) | | - | - | 2997 (32.7) | - | 2739 (31.2) | 258 (69.5) | <0.001 |
| Age at first cannabis use | | - | - | 20.0 [18.0-24.0] | - | 20.0 [18.0-24.0] | 20.0 [18.0-24.5] | 0.037 |
| Early onset (initial cannabis use prior to 17 years of age) | | - | - | 954 (13.7) | - | 886 (13.3) | 68 (22.6) | <0.001 |
| Duration of use, y | | - | - | 10.7 ± 13.1 | - | 9.3 ± 11.3 | 41.2 ± 11.4 | <0.001 |
| Long duration of use (duration of use was >5 years) | | - | - | 3504 (52.8) | - | 3217 (50.8) | 287 (97.0) | <0.001 |
| Duration of cannabis use abstinence | | - | - | - | - | 38.0 [25.9-43.0] | - | - |
| Duration between cognitive function measures, years | | 5.7 ± 2.0 | 5.8 ± 2.0 | 5.6 ± 2.1 | <0.001 | 5.5 ± 2.1 | 6.7 ± 1.1 | <0.001 |

Continuous values are reported as Mean ±SD or Median [q1-q3] and dichotomous values are reported as N (%).
Obesity was defined as body mass index ≥30; Physical activity was defined as number of days per week of moderate physical activity >10 min; Current use defined if they had used cannabis within the year before the initial cognitive assessment; Regular use defined as use of cannabis more than once a month; Early onset was defined as reported initial cannabis use prior to 17 years of age, late onset was defined as reported initial cannabis use at age 17 or later; Long duration of use was defined when duration of use was >5 years.

**Supplement Table 3.** Results from multivariable regression analyses showing the associations of pattern of cannabis use with change in cognitive function over time

|  | Attention  B (SE) N=46,119 | Executive function  B (SE)  N=39,992 | Processing speed  B (SE) N=51,147 |
| --- | --- | --- | --- |
| **Panel A – comparing ever use to no use** | | | |
| No use | 0 (ref.) | 0 (ref.) | 0 (ref.) |
| Ever Use | -0.001 (0.004)  P=0.919 | **0.008 (0.004)**  **P=0.030** | 0.060 (0.060)  P=0.321 |
| **Panel B – comparing former use and current use to never use** | | | |
| No use | 0 (ref.) | 0 (ref.) | 0 (ref.) |
| Former use | -0.001 (0.004)  P=0.910 | **0.008 (0.004)**  **P=0.030** | 0.063 (0.061) p=0.305 |
| Current use | 0.0001 (0.016)  P=0.961 | 0.004 (0.016)  P=0.774 | -0.015 (0.263)  P=0.952 |
| **Panel C – comparing current use to former use** | | | |
| Former use | 0 (ref.) | 0 (ref.) | 0 (ref.) |
| Current use | 0.003 (0.015)  P=0.821 | -0.004 (0.016)  P=0.808 | 0.139 (0.258) P=0.590 |

SE, Standard error; Attention, Trail Making A; Executive function, Trail Making B; Processing speed, Symbol digit; Current use defined if they had used cannabis within the year before the initial cognitive assessment.

Models adjusted for age, sex, education, socioeconomic status, tobacco smoking, alcohol use, lifetime substance use addiction, obesity, physical activity and history of ischemic heart disease, hypertension, diabetes, first cognitive function measure and time between two cognitive function measures. Bold values indicate statistical significance (p<0.05)

**Supplement Table 4.** Results from multivariable regression analyses showing the associations of pattern of cannabis use with change in cognitive function over time

| Pattern of use | | Attention  B (SE) | Executive function  B (SE) | Processing speed  B (SE) |
| --- | --- | --- | --- | --- |
| Former users | N | 9,999 | 9,943 | 10,837 |
|  | Less than once a month | 0 (ref.) | 0 (ref.) | 0 (ref.) |
|  | Once a month or more, but not every week | -0.003 (0.009)  P=0.817 | -0.004 (0.009)  P=0.654 | -0.003 (0.160) P=0.798315 |
|  | Once a week or more, but not every day | -0.005 (0.008)  P=0.535 | 0.0101 (0.009)  P=0.243 | 0.019 (0.145) P=0.892 |
|  | Every day | 0.007 (0.015)  P=0.653 | 0.026 (0.016)  P=0.103 | -0.421 (0.267) P=0.115 |
|  | Not regular use | 0 (ref.) | 0 (ref.) | 0 (ref.) |
|  | Regular use | -0.003 (0.006)  P=0.691 | 0.006 (0.007)  P=0.341 | -0.041 (0.112) P=0.715 |
|  | Early onset | 0 (ref.) | 0 (ref.) | 0 (ref.) |
|  | Late onset | -0.011 (0.010)  P=0.283 | 0.009 (0.011)  P=0.387 | 0.105 (0.174) P=0.543 |
|  | Initial cannabis use age | 0.0005 (0.0004)  P=0.288 | 0.0002 (0.0004)  P=0.742 | 0.007 (0.007) P=0.375 |
|  | Short duration | 0 (ref.) | 0 (ref.) | 0 (ref.) |
|  | Long duration | -0.012 (0.007)  P=0.073 | -0.014 (0.007)  P=0.056 | **-0.248 (0.118) P=0.035** |
|  | Duration of use | -0.0001 (0.0003)  P=0.779 | -0.0001 (0.0001)  P=0.370 | **-0.019 (0.005) P<0.001** |
| Current users | N | 373 | 328 | 374 |
|  | Less than once a month | 0 (ref.) | 0 (ref.) | 0 (ref.) |
|  | Once a month or more, but not every week | 0.050 (0.056)  P=0.372 | -0.033 (0.056)  P=0.552 | 0.713 (0.819) P=0.385 |
|  | Once a week or more, but not every day | -0.026 (0.045)  P=0.561 | -0.043 (0.044)  P=0.322 | 0.412 (0.676) P=0.543 |
|  | Every day | -0.038 (0.047)  P=0.415 | -0.065 (0.046)  P=0.164 | 0.023 (0.710) P=0.974 |
|  | Not regular use | 0 (ref.) | 0 (ref.) | 0 (ref.) |
|  | Regular use | -0.013 (0.037)  P=0.725 | -0.048 (0.037)  P=0.188 | 0.352 (0.562) P=0.532 |
|  | Early onset | 0 (ref.) | 0 (ref.) | 0 (ref.) |
|  | Late onset | 0.039 (0.051)  P=0.446 | -0.006 (0.051)  P=0.910 | -0.563 (0.773) P=0.466 |
|  | Initial cannabis use age | -0.0006 (0.002)  P=0.779 | -0.001 (0.001)  P=0.526 | -0.003 (0.026) P=0.911 |
|  | Duration of use | 0.0001 (0.002)  P=0.778 | 0.0001 (0.002)  P=0.602 | 0.003 (0.026) P=0.892 |

SE, Standard error; Attention, Trail Making A; Executive function, Trail Making B; Processing speed, Symbol digit; Current use defined if they had used cannabis within the year before the initial cognitive assessment; Regular use defined as use of cannabis more than once a month; Early onset was defined as reported initial cannabis use prior to 17 years of age, late onset was defined as reported initial cannabis use at age 17 or later; Long duration of use was defined when duration of use was >5 years.

Models adjusted for age, sex, education, socioeconomic status, tobacco smoking, alcohol use, lifetime substance use addiction, obesity, physical activity and history of ischemic heart disease, hypertension, diabetes, first cognitive function measure and time between two cognitive function measures. Bold values indicate statistical significance (p<0.05)
